# Plasmonic Chiral Metasurfaces for Real‐Time Refractive Index Sensing and In Situ Monitoring of Thin Film Growth

**DOI:** 10.1002/smsc.202500568

**Published:** 2026-03-16

**Authors:** Sevil Veysalova, Benjamin Boglio, François Courtier, Gero Decher, Olivier Felix, Matthias Pauly

**Affiliations:** ^1^ Université de Strasbourg, CNRS Institut Charles Sadron UPR 22 Strasbourg France; ^2^ ENS de Lyon CNRS LCH, UMR 5182 Lyon France

**Keywords:** chiral nanostructures, circular dichroism, metasurfaces, plasmonics, refractive index sensing, silver nanowire assemblies

## Abstract

Molecular sensing plays a crucial role in biomedical, chemical, and environmental applications. Traditional plasmonic sensors based on surface plasmon resonance offer excellent sensitivity but face limitations in molecular specificity, scalability, and real‐time operation. Here, we present a novel plasmonic chiral metasurface sensor fabricated via a scalable bottom‐up approach using Layer‐by‐Layer (LbL) assembly and grazing incidence spraying of silver nanowires, and which exhibits a strong circular dichroism (CD) signal. Once integrated into a microfluidic flow cell, the sensor enables real‐time spectroscopic measurements with high stability, reproducibility, and reusability. Refractive index sensing reveals a sensitivity based on the wavelength of the peak in the CD spectra of ∼56 nm·refractive index unit (RIU)^−1^ and a sensitivity based on the CD intensity at a fixed wavelength of ∼18 700 mdeg·RIU^−1^, yielding a limit of detection as low as 1.6 × 10^−4^ RIU, i.e. 20 times lower than the conventional extinction‐based methods. Moreover, the device enables in situ monitoring of polymer adsorption throughout the LbL assembly process, thereby providing a highly sensitive and label‐free method for investigating interfacial phenomena. The present work establishes a robust and scalable platform for optical sensing by integrating the distinct advantages of chiral plasmonic materials with real‐time fluidic control and cost‐effective fabrication methodologies.

## Graphical abstract

A sensor based on a plasmonic chiral metasurface is fabricated via Layer‐by‐Layer assembly and grazing incidence spraying of silver nanowires, and integrated in a flow‐cell. It enables stable, real‐time circular dichroism measurements with high sensitivity toward refractive index changes of the surroundings. The device allows in situ, label‐free monitoring of interfacial processes, offering a robust, low‐cost platform for optical sensing.

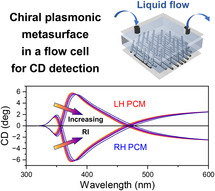

## 1 Introduction

Molecular sensing constitutes a fundamental component of a wide range of scientific and technological disciplines, including environmental monitoring, biomedical diagnostics, food safety, and chemical processing. With the increasing demand for real‐time, label‐free, and highly sensitive detection methodologies, optical sensors have emerged as indispensable analytical tools [1]. Among these, sensors based on surface plasmon resonance (SPR) and localized surface plasmon resonance (LSPR) have garnered significant interest owing to their capacity to confine electromagnetic radiation beyond the diffraction limit and to induce substantial enhancement of local electromagnetic fields [2, 3]. SPR arises from the excitation of surface plasmon polaritons, which are propagating collective electron charge density oscillations occurring at metal–dielectric interfaces, whereas LSPR is associated with electron oscillations confined to subwavelength metallic nanostructures. Both phenomena enable label‐free and real‐time monitoring by detecting variations in the optical response, such as resonance frequency shifts, originating from local refractive index (RI) changes upon analyte adsorption. This mechanism provides exceptionally high sensitivity and has been demonstrated to allow detection down to the single‐molecule scale [4].

Recent progress in nanophotonics and materials science has facilitated the development of plasmonic chiral metasurfaces (PCMs) [5, 6] as promising alternatives to conventional plasmonic sensing platforms [7, 8, 9, 10]. These metasurfaces are deliberately engineered nanostructures with broken geometric symmetry, specifically designed to exhibit differential interactions with circularly polarized light. Such interactions give rise to pronounced circular dichroism (CD) responses, manifested as the differential absorption of left‐ and right‐handed circularly polarized light. In 2010, Hendry et al. reported the first chiral metasurface for molecular sensing, using a single‐layer gammadion structure to detect various proteins by measuring the spectral shift in the far‐field spectrum caused by the near‐field interactions between the chiral molecule and the metasurface [11]. Zhao et al. explored the use of twisted plasmonic metamaterials to sense molecular chirality by enhancing chiral optical signals using near‐field interactions and eliminating background interference [12]. More recently, Feng et al. introduced twisted‐stacked silver nanowire arrays that exhibit CD signals at interband transitions and plasmonic extinction regions, enabling greater sensitivity and reliability in chiral biosensing [13].

However, such PCMs have mostly been fabricated using top‐down technologies such as direct laser writing [14], focused ion beam processing [15], or e‐beam lithography [16]. These methods, while highly precise, are inherently costly, time‐consuming, and unsuitable for large‐area or scalable production. To address these limitations, bottom‐up fabrication strategies based on the self‐assembly of wet‐synthesized, well‐defined building blocks have been proposed as a promising approach to fabricate chiral metamaterials at macroscopic scales [17, 18, 19]. In particular, our group has previously demonstrated that tunable PCMs can be realized by stacking multiple layers of aligned silver nanowires (AgNWs), where each successive layer is rotated relative to the previous one to introduce chirality [20, 21, 22]. We employ grazing incidence spraying (GIS) [23, 24] to deposit oriented AgNW layers. The in‐plane alignment of AgNWs is induced by the shear flow of the flowing liquid and leads to the formation of a monolayer of nanowires pointing in the spray direction. Layer‐by‐Layer (LbL) assembly [25, 26] is used to construct multilayer architectures, allowing for the control of the sequence of deposited materials. The main advantage of this combined approach is that the orientation can be independently chosen for each AgNW layer. This bottom‐up strategy yields PCMs with giant CD signals that can be tuned by adjusting the nanowire density, interlayer angle, and spacing [21]. The high CD is obtained from the stacking of layers with a uniaxial optical anisotropy (i.e. displaying linear birefringence and dichroism) with a certain spacing and a twisting angle between the optical axis of each NW layer. The maximum CD is obtained when the angle between the two layers is 45°.

In this study, chiral metasurfaces are integrated into a custom‐engineered microfluidic flow cell, which facilitates real‐time CD measurements in direct contact with solutions of varying refractive indices. The sensitivity of the system to RI variations is assessed by tracking the spectral shifts of CD features as a function of the glycerol‐to‐water concentration ratio. Furthermore, the platform demonstrates the capability to monitor the in situ and real‐time growth ofpolymer thin films, thereby providing a robust, label‐free optical approach for the investigation of interfacial assembly processes.

## 2 Results and Discussion

### 2.1 PCM Fabrication and Integration into a Flow Cell System

**FIGURE 1:**
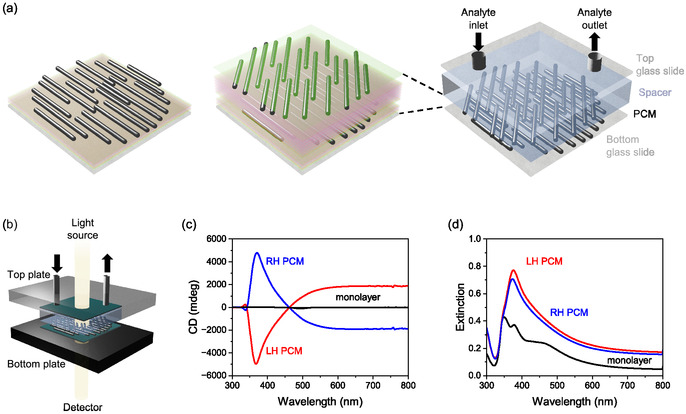
Fabrication of the PCM and its integration into a flow cell system. (a) A first layer of aligned AgNWs is deposited using GIS onto a substrate coated with a polyelectrolyte multilayer (PEI/PSS/PAH/PSS/PEI). A dielectric spacer composed of a polyelectrolyte multilayer (PEI/(PSS/PAH)_5_/PEI) is then added, followed by deposition of a second layer of aligned AgNWs oriented at ±45° relative to the first AgNW layer, and finally a top layer of PEI. (b) The resulting PCM structure is incorporated into a flow cell, which is placed in a holder allowing light to pass through a 5 mm diameter circular aperture. (c) CD and (d) extinction spectra are shown for an oriented AgNW monolayer, as well as for left‐ and right‐handed two‐layer PCMs (LH PCM and RH PCM, respectively). For extinction measurements, the incident light is linearly polarized perpendicular to the long axis of the nanowires in the monolayer or in the bottom layer of the PCM, selectively exciting the transverse plasmon resonance mode.

A PCM was fabricated by combining LbL assembly and GIS, an approach previously developed in our group for preparing complex (e.g. helical) multilayer films in which the composition and orientation can be controlled independently in each layer [21, 24]. The overall fabrication strategy and flow cell integration are illustrated in Figure 1. Briefly, polyelectrolyte multilayers (PEM) were deposited via LbL assembly to serve both as an adhesion layer on the substrate and as a dielectric spacer between successive layers of AgNWs. The polyelectrolytes used in the PEMs were poly(ethyleneimine) (PEI), poly(sodium‐4‐styrenesulfonate) (PSS), and poly(allylamine hydrochloride) (PAH). The substrate was initially coated with a PEI/PSS/PAH/PSS/PEI precursor film with a thickness of ∼5 nm. The first layer of unidirectionally aligned AgNWs was then deposited using GIS (Figure 1a), resulting in an anisotropic film characterized by a 2D nematic order parameter of ∼0.85. A second PEM spacer layer with a (PEI/(PSS/PAH)_5_/PSS/PEI) architecture and a thickness of ∼13 nm was subsequently assembled. A second AgNW layer was then deposited by GIS at an angle of either + 45° or −45° relative to the first AgNW layer, yielding a left‐ or right‐handed structure, respectively. A final PEI layer was added to enhance the stability of the assembly and avoid desorption of the top AgNW layer (Figure 1a).

The resulting PCMs exhibit strong CD in the UV‐visible range, with CD intensities reaching up to ±4000 mdeg and corresponding g‐factors up to 0.4 (Figure 1c). The maximum CD signal is observed at 385 nm, and the CD spectra are mirror‐symmetric for left‐ and right‐handed enantiomorphs. Extinction spectra show an absorption peak at 378 nm, attributed to the transverse LSPR of AgNWs (Figure 1d). For comparison, a monolayer of aligned AgNWs, being achiral, exhibits no CD response.

The PCM was integrated into a custom‐designed flow cell (Figure 1a), held in place by a home‐made holder allowing transmission measurements through a 5 mm diameter aperture (Figure 1b and Figure S1). The flow cell confines *a* < 200 µm‐thick solution layer in contact with the PCM, enabling spectroscopic measurements in transmission at a fixed location. Fixed sample positioning is crucial, as minor misalignments can induce spectral variations due to local heterogeneity in AgNW orientation or density. This design significantly improves the reproducibility and reliability of both CD and UV–vis–NIR extinction measurements and facilitates comparative analysis across different solution compositions. The density of AgNWs is different for the different samples used throughout this study, and thus CD spectra reported here typically range from ±2 000 to ±10 000 mdeg. The performance and reliability of the flow cell were validated through assessments of structural stability, sensitivity, and reusability of the PCM sensor.

### 2.2 PCM Stability

**FIGURE 2:**
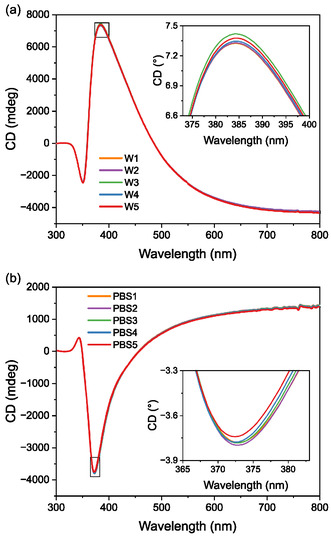
Structural stability of the PCM. CD spectra of (a) a RH PCM subjected to repeated rinsing with Milli‐Q water (W), and (b) a LH PCM subjected to repeated rinsing with PBS. In both cases, the CD response remains consistent, indicating structural integrity of the metasurface under aqueous and saline conditions. The black rectangles indicate the area that is zoomed in the insets.

The structural stability of the PCM was evaluated using repeated rinse‐and‐measure experiments in Milli‐Q water and phosphate‐buffered saline (PBS, 0.01 M phosphate buffer, 0.0027 M potassium chloride, and 0.137 M sodium chloride, pH 7.4). These tests were designed to assess whether the chiral structure remains stable upon repeated exposure to aqueous and moderately ionic environments, which are known to potentially weaken electrostatic interactions that stabilize PEMs [27]. In each experiment, the flow cell was filled with either water or PBS, and the CD spectrum was measured. The solution was then replaced with a fresh liquid of the same type, and the CD spectrum was measured again. This rinse‐and‐measure cycle was repeated five times for a right‐handed (RH) PCM in water (Figure 2a) and for a left‐handed (LH) PCM in PBS (Figure 2b), with highly consistent CD spectra observed in both cases. Maximum CD values remained stable within 0.6% at 7360 ± 40 mdeg ($\lambda_{\max\;CD}=384\;nm$) and −3780 ± 22 mdeg ($\lambda_{\max CD}=373\;nm$), respectively.

The measurements confirm that the PCMs retain their structural integrity under repeated rinsing in both aqueous and saline environments. As 5 mL of each solution was injected in less than 5 s, this shows that the PCM is stable and that the flow cell does not leak, even under high shear flow. It also demonstrates that no contaminants diffuse from the flow cell elements (in particular from the sealing gasket) to the analyte medium. Consequently, the spectral variations observed upon exposure to different analytes can be ascribed to specific solution–surface interactions, rather than to degradation or restructuring of the metasurface itself. Furthermore, comparative measurements revealed CD spectral variations of similar magnitude when the sample was left in the instrument and recorded multiple times without rinsing. This observation suggests that the minor fluctuations originated predominantly from instrumental variability, thereby corroborating the robustness and stability of the PCM platform during sensing experiments.

### 2.3 RI Sensing

**FIGURE 3:**
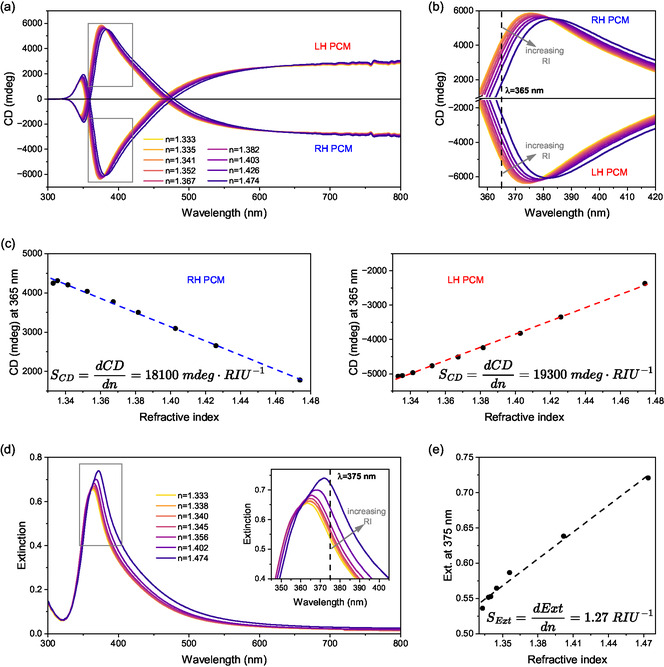
RI sensing using CD and extinction measurements. (a) CD spectra of RH and LH PCMs in contact with aqueous glycerol solutions of varying RI. (b) Magnified view of the CD spectra highlighting the red shift; the dashed line indicates a fixed wavelength at *λ *= 365 nm. (c) CD intensity at *λ* = 365 nm plotted as a function of RI for both PCM enantiomorphs, with the slope corresponding to the CD sensitivity $S_{CD}$ at 365 nm. (d) Extinction spectra of a monolayer of oriented AgNWs recorded under light linearly polarized perpendicular to the alignment direction, selectively exciting the transverse LSPR mode, in contact with media of varying RI, (e) Extinction at *λ *= 375 nm as a function of RI with the slope corresponding to the extinction sensitivity 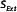 at 375 nm.

The sensitivity of the PCM to variations in the RI of the surrounding medium was evaluated by measuring its CD spectra in contact with mixtures of water and glycerol at varying concentrations (Figure 3a). These mixtures enabled modulation of the RI from $n_{w}=1.333$ (pure water) to $n_{gly}=1.474$ (pure glycerol). The RI of intermediate mixtures was calculated from $n=A_{n}C+n_{D}^{*}$, where n is the RI of the intermediate mixture, $A_{n}\left(\frac{L}{mol}\right)$ is an experimental parameter which is $0.0105\frac{L}{mol}$ for glycerol‐water mixtures, C is the molar concentration of glycerol, and $n_{D}^{*}$ is the RI of the solvent (1.333 for water) (Table S1) [28]. As expected, increasing the RI induced a redshift of the CD peak (Figure 3b). For the LH PCM, the wavelength of the peak in the CD spectra shifted from 374 nm in water to 381.5 nm in glycerol. A similar shift from 375.5 nm to 383.5 nm was observed for the RH PCM. The spectral shift as a function of RI is linear (Figure S2a), yielding a peak CD wavelength sensitivity ($S_{\lambda_{\max CD}}=\frac{d\lambda_{\max CD}}{dn}$) of 55.7 nm per refractive index unit (RIU) averaged from both enantiomorphs.

For comparison, the RI sensitivity of a monolayer of aligned AgNWs was evaluated via polarized UV–vis–NIR extinction spectroscopy, with the incident light polarized perpendicular to the nanowire alignment to selectively excite the transverse LSPR (Figure 3d). A redshift in the extinction maximum was observed, from 364 nm in water to 372 nm in glycerol, corresponding to a sensitivity of $S_{\lambda_{\max Ext}}=\frac{d\lambda_{\max Ext}}{dn}=53.5\;nm\cdot RIU^{-1}$ (Figure S2b). Therefore, the RI sensitivities based on the shift of the peak wavelength of CD spectra and extinction spectra are very close.

In addition to tracking peak shifts, sensitivity was also assessed by measuring changes in CD and extinction at a fixed wavelength. The CD sensitivity at *λ *= 365 nm was measured for both PCM enantiomorphs, yielding an averaged value of $S_{CD}=\frac{dCD}{dn}\approx 18\;700\;mdeg\cdot RIU^{-1}$ (Figure 3c). This wavelength was selected as it offers the highest sensitivity (Figure S3). A similar trend was observed for PCMs with a thicker polyeletrolyte spacer between the AgNW layers (Figure S4). The sensitivity measured from the extinction value at a fixed wavelength of *λ *= 375 nm was $S_{Ext}=\frac{dExt}{dn}\approx 1.27\;RIU^{-1}$ (Figure 3e).

Additionally, the CD spectra of the PCM were simulated as a function of the surrounding RI using a 4 × 4 transfer matrix formalism (section 3 in supporting information). This formalism rigorously quantifies the change in amplitude and phase of light across each layer of a multilayer stack. The simulated stack replicated the experimental conditions (Figure S5), and the simulation captured the main CD features and their dependence on ambient RI (Figure S6).

To put the sensitivity values in perspective, the detection limit–in terms of the minimum change of RI that can be detected–was calculated based on the intrinsic spectral resolution of the respective spectrometers (*σ*), measured as the standard deviation of repeated measurements in a constant environment. The limit of detection (LOD) of the RI was determined as $LOD=\frac{3\sigma }{S}$ (Table 1).

**TABLE 1:** Comparison of RI sensing metrics based on extinction and CD measurements.

	Sensitivity S	Standard deviation *σ*	Limit of detection LOD
$\lambda_{\max Ext}$	$53.5\;nm\cdot RIU^{-1}$	0.054 nm	$3.0\times 10^{-3}\;RIU$
$\lambda_{\max\;CD}$	$55.7\;nm\cdot RIU^{-1}$	0.15 nm	$8.1\times 10^{-3}\;RIU$
*Extinction at* λ=375 nm	$1.27\;RIU^{-1}$	$4.0\times 10^{-4}$	$9.4\times 10^{-4}\;RIU$
*CD at* λ=365 nm	$18\;700\;mdeg\cdot RIU^{-1}$	1.0 mdeg	$1.6\times 10^{-4}\;RIU$

These results demonstrate that RI sensing based on changes of the CD at a fixed wavelength offers the highest sensitivity and lowest LOD among the evaluated metrics. Notably, the LOD achieved using CD at *λ *= 365 nm is approximately six times lower than that obtained using extinction at *λ* = 375 nm, and roughly twenty times lower than that obtained by monitoring the $\lambda_{max}$ shift in extinction spectra, an approach commonly employed for nonchiral SPR sensors.

Previous studies on RI sensing based on chiral nano‐objects have shown that sensitivity of a few hundreds $nm\cdot RIU^{-1}$ can be reached with very well‐defined samples prepared by top‐down approaches [15, 29], but the comparison with the values reported here remain difficult as many different approaches are used in the literature to quantify the spectral changes (shift of the wavelength of maximum extinction or CD spectra [15], shifts in the zero‐crossing point of CD spectra [29], shifts of the 90°‐crossing points of the optical rotation [30], ratio of major/minor axis of transmitted elliptically polarized light of chiral plasmonic structure arrays [31]).

### 2.4 PCM Reusability

**FIGURE 4:**
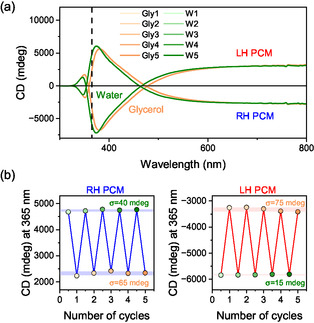
Reusability of the PCM‐based RI sensor. (a) CD spectra recorded over five cycles of alternated exposure to water (W) and glycerol (Gly). (b) CD intensity at *λ *= 365 nm for both RH and LH PCMs, confirming signal reproducibility.

Sensor reusability is a critical parameter for practical deployment. To assess this, both LH and RH PCM sensors were subjected to five consecutive cycles of alternated exposure to water and glycerol (Figure 4a). The CD spectra collected during each cycle showed clear and reproducible differentiation between the two solutions, with consistent CD values observed across all repetitions (Figure 4b). For the RH PCM, the standard deviation of the CD signal was 40 mdeg in water and 65 mdeg in glycerol. In the case of the LH PCM, the standard deviations were 15 mdeg and 75 mdeg for water and glycerol, respectively. These low variations (0.05% for water, 0.2% for glycerol) highlight the excellent reproducibility and robustness of the PCM under repeated use.

### 2.5 PCM for Real‐Time Monitoring of LbL Assembly

To further demonstrate the utility of the PCM‐based sensor, we monitored the polyelectrolyte LbL assembly in real‐time and in situ in the microfluidic flow cell. The experiment mimicked conventional LbL deposition conditions, where substrates are alternately exposed to solutions of oppositely charged polymers. Each adsorption step involved flowing a polyelectrolyte solution through the chamber and allowing 15 min of contact time, followed by three rinsing cycles (2 min each) with Milli‐Q water before proceeding with the next layer. PEI was employed as the initial layer, followed by alternating deposition of PSS and PAH to build the PEM.

**FIGURE 5:**
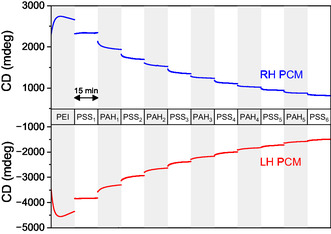
In situ and real‐time monitoring of polyelectrolyte multilayer assembly in a microfluidic flow cell. CD intensity at *λ *= 365 nm during the sequential deposition of PEI, PSS, and PAH. Rinsing steps between depositions are omitted for clarity, full trace is provided in Figure S7.

The CD at *λ *= 365 nm was continuously monitored throughout the LbL film build‐up (Figure 5). Rinsing steps are omitted in Figure 5 for clarity, but are included in Figure S7. The absolute value of the CD increased upon adsorption of the first PEI layer, and decreased for the subsequent PSS and PAH layers, which is consistent with the full CD spectra measured for the first layers (Figure S8). As the multilayer assembly progressed, the incremental CD change per layer diminished. This trend is attributed to the exponential decay of the plasmonic electric field away from the PCM surface [32], which lowers the sensitivity to adsorbed layers as the thickness increases. The CD signal reached a plateau after five polyelectrolyte layer pairs (approximately 15 nm total thickness), consistent with literature values for the decay length of the electric field around plasmonic nanoparticles [33]. SEM images taken after deposition of polyelectrolyte layers confirm that the PEM deposits on top of the PCM without affecting its structure (Figure S9).

The time‐resolved CD measurements also revealed the typical adsorption kinetics of the polyelectrolytes, and a deposition time of ∼10 min per layer. The variation with time of the intensity of CD can be fitted to a double exponential (Figure S10), consistent with the adsorption kinetics measured by a SPR sensor or quartz crystal microbalance (QCM) [34].

Real‐time monitoring of LbL assembly has relied on techniques such as QCM [35] and SPR techniques [36]. Our CD‐based method provides a complementary approach, offering several advantages: high sensitivity, low sample volume requirements, real‐time feedback, and sensor reusability. While direct quantitative comparison across techniques is challenging due to differing measurement principles (e.g., frequency shifts in QCM, angle changes in SPR), the high sensitivity of our method to changes in optical activity and local RI offers a promising alternative for characterizing nanoscale interfacial processes.

## 3 Conclusion

In conclusion, we have developed a PCM sensor fabricated through a scalable bottom‐up approach and integrated into a microfluidic flow cell. The platform exhibits excellent structural stability, high sensitivity to RI variations, strong reproducibility across multiple measurements, and reusability over successive sensing cycles. The RI sensing performance is characterized by a peak CD wavelength sensitivity of approximately 56 nm·RIU^−1^ and a fixed‐wavelength CD intensity sensitivity of about 18,700 mdeg·RIU^−1^, corresponding to a detection limit as low as 1.6 × 10^−4^ RIU, nearly twenty times better than conventional extinction‐based techniques. These results establish fixed‐wavelength CD as a highly effective metric for low‐concentration analyte detection. Moreover, the sensor demonstrated the capability to monitor polyelectrolyte multilayer growth in real time and in situ, underscoring its potential for studying interfacial processes. Such a sensor could further be rendered specific to a given analyte by functionalizing the AgNWs with a molecule that binds specifically to the targeted analyte. In combination with its cost‐effective fabrication, the performance demonstrated here highlights the benefit of this platform in widespread practical optical sensing applications in the fields of biosensing or environmental monitoring.

## 4 Materials and Methods

### 4.1 Materials

Poly(ethyleneimine) (PEI, $M_{W}∼25,000$ g⋅ mol^−1^; CAS 9002‐98−6), poly(sodium‐4‐styrenesulfonate) (PSS, $M_{W}∼70,000$ g⋅ mol^−1^; CAS 25 704‐18−1), poly(allylamine hydrochloride) (PAH, $M_{w}∼50,000$ g⋅ mol^−1^, CAS 71 550‐12−4), phosphate buffered saline (PBS, tablet, pH 7.2–7.6) were purchased from Sigma–Aldrich. Glycerol (anhydrous, ≥ 99.5%, CAS 56‐81−5) was purchased from VWR. Silver nanowires (AgNWs, A30 (d ≈ 30 nm, L ≈ 30 μm)), (suspension in isopropanol at 10 mg·mL^−1^) were purchased from Novarials Corporation. Glass slides (24 mm × 24 mm, thickness 1 mm) were purchased from Thermo Scientific. All the chemicals were used without further purification. All solutions were prepared with ultrapure Milli‐Q water produced by a Milli‐Q Advantage A10 water purification system (Merck–Millipore).

### 4.2 Substrates

Prior to any modification, glass substrates were thoroughly cleaned. They were immersed in a 0.1 M NaOH solution for 15 min. Substrates were then rinsed with Milli‐Q water and placed in a 50:50 (v/v) mixture of ethanol and Milli‐Q water for 15 min. They were subsequently rinsed with Milli‐Q water and dried using compressed air. Finally, the glass slides were cleaned/activated by 3 min plasma treatment in a plasma cleaner (Harrick Plasma, PDC‐002, USA) at a high RF power (∼30 W).

### 4.3 Fabrication of PCM

PEM were deposited on cleaned substrates using dipping‐ and spray‐assisted LbL assembly. A total of five layers were deposited in the following sequence before adding the first monolayer of AgNWs: PEI/PSS/PAH/PSS/PEI.

The PEI layers were applied using the dipping method. The substrate was immersed in a PEI solution (2.5 mg·mL^−1^ in Milli‐Q water) for 15 min. After PEI deposition, the substrate was rinsed three times with Milli‐Q water for 2 min and dried with compressed air. PSS and PAH layers were deposited using a spray‐assisted LbL method. Spraying was carried out perpendicularly to the receiving surface, which was fixed vertically. The polyelectrolyte solution was sprayed on the substrate for 5 s, left to stand for 5 s and then rinsed by spraying Milli‐Q water for 10 s. The substrate was then dried with compressed air. PSS solution was prepared at a concentration of 0.616 mg·mL^−1^ in 0.5 M NaCl, while PAH solution was prepared at a concentration of 0.077 mg·mL^−1^ in 0.5 M NaCl. After depositing the polyelectrolyte layers, the first monolayer of AgNWs was added using GIS. Commercial suspension containing 1 g of AgNWs in 100 mL of isopropanol was diluted 30–50 times in Milli‐Q water. The diluted AgNW suspension was sprayed for 200 s at a flow rate of 1 and 2 mL·min^−1^. The sample was then rinsed with Milli‐Q water for 20–40 s.

After forming the first monolayer of AgNWs, another polyelectrolyte multilayer was deposited, with the following sequence: PEI/(PSS/PAH)_5_/PSS/PEI. The PEI layers were deposited by dipping, and the PSS/PAH layers by spraying under the experimental conditions described previously. The second AgNW monolayer was added using GIS by spraying the AgNW suspension at *a* + 45° angle for LH chiral metasurfaces and a −45° angle for RH ones with respect to the AgNW orientation in the first monolayer. Finally, a layer of PEI was added by dipping.

### 4.4 Integration of PCM within a Flow Cell

The PCM was integrated into a flow cell using a home‐made holder (Figure S1). This was achieved by adding a silicone gasket of approximately 200 µm and a cover made from a glass slide with two pre‐drilled holes on top of the substrate functionalized with the PCM. The different parts of the flow cell were held together by the upper and lower support plates of the holder, which were screwed together. Tube fittings were screwed directly to holes drilled in the upper support plate and equipped with tubing for solvent exchange using a syringe.

Additionally, when we place the assembly in the home‐made holder, the top and bottom plates exert pressure that compresses the silicone gasket to avoid leakage. We believe that this reduces the gap to less than 200 µm, resulting in the solution layer in contact with the PCM.

### 4.5 CD Spectroscopy

CD spectra were measured with a Jasco J‐1700 CD Spectrometer in the spectral range between 300 and 800 nm. Before each measurement, a baseline was measured on the flow cell equipped with two glass slides with a 200 µm spacer in air. A data pitch of 0.5 nm, a bandwidth of 1 nm, a data integration time of 2 sec, and a scanning speed of 100 nm·min^−1^ were used for the measurements.

### 4.6 UV–Vis–NIR Spectroscopy

Extinction spectra were recorded using a Cary 5000 UV–vis–NIR spectrophotometer (Agilent Technologies) in the 300–800 nm wavelength range. Measurements were performed at a scan rate of 600 nm·min^−1^. Baseline correction was carried out using the flow cell equipped with two glass slides with a 200 µm spacer in air. The instrument was operated with the polarizer oriented at 90° with respect to the bottom AgNW layer.

## Supporting Information

Additional supporting information can be found online in the supporting information section. **Supporting** **Fig.** **S1:** (a) Exploded view of the different elements constituting the flow cell and its holder. (b) Scheme of the light path through the flow cell and PCM. (c) Photo of the flow cell and holder. **Supporting Fig. S2**: RI Sensing based on the shift of the peak of (a) CD and (b) extinction spectra. **Supporting Fig. S3**: RI sensing using CD. (a) CD spectra of RH and LH PCMs in contact with aqueous glycerol solutions of varying RI. (b, c) CD intensity at λ = 350 nm, λ = 365 nm, λ = 440 nm and λ = 500 nm (from top to bottom) as a function of RI for (b) RH PCM and (c) LH PCM. **Supporting Fig. S4**: RI sensing using CD spectra for a PCM with a thicker polyelectrolyte spacing between the AgNW layers. (a) CD spectra of left handed PCMs in contact with aqueous glycerol solutions of varying RI. (b) Magnified view of the CD spectra (a). (c) λ_max_ as a function of RI. CD intensity at (d) λ = 355 nm and (e) λ = 388 nm plotted as a function of RI for the LH PCM. **Supporting Fig. S5**: Schematic of the simulated multilayer stack: aqueous/glycerol entry medium with varying RI (n_var_ = 1.333, …, 1.474), oriented AgNW film (±45°, 30 nm), polyelectrolyte spacer (13 nm, n_var_), oriented AgNW film (0°, 30 nm), polyelectrolyte spacer (5 nm, n = 1.465), and quartz substrate. **Supporting Fig. S6**: (a) Experimental (top) and simulated (bottom) CD spectra for refractive indices from 1.333 (yellow) to 1.474 (purple). Dashed line indicates the wavelength used for sensitivity analysis (365 nm experimentally and 350 nm for the simulations) (b) CD at aforementioned wavelength versus RI, linear fits yield slopes with magnitudes of ∼18100 mdeg·RIU^−1^ (exp., R² = 0.997) and ∼865 mdeg·RIU^−1^ (sim., R² = 0.999). **Supporting Fig. S7:** In situ assembly of PEM in the flow cell with rinsing steps. The CD is monitored at λ = 365 nm throughout the deposition of polyethyleneimine (PEI), poly(sodium 4‐styrenesulfonate) (PSS), and poly(allylamine hydrochloride) (PAH). **Supporting Fig. S8**: CD spectra of in situ assembly of PEM in the flow cell. The spectra labeled “PCM” is the one of the PCM in air before adding the polyelectrolyte layers, while the other spectra are measured after adsorption of the respective polyelectrolyte layers, the PCM still in contact with the polyelectrolyte solution. The inset shows a zoom of the CD spectra for LH PCM. **Supporting** **Fig.** **S9:** Cross‐section SEM images of (a) PCM, (b) PCM+PEI, (c) PCM+PEI/(PSS/PAH)_7_, (d) PCM+PEI/(PSS/PAH)_10_, (e) PCM+PEI/(PSS/PAH)_15_. **Supporting Fig. S10:** In situ assembly of PEM in the flow cell without the rinsing steps. The measured CD as function of time (black points) is fitted with a double exponential growth curve (colored line).^[1]^ $CD=A+K_{1}\left(1‐\exp\left(‐\frac{1‐t_{D}}{\tau_{1}}\right)\right)+K_{2}\left(1‐\exp\left(‐\frac{1‐t_{D}}{\tau_{2}}\right)\right)$for the first PAH layer (from 30 to 45 min), the second PSS layer (from 45 to 60 min) and the second PAH layer (from 60 to 75 min). The data are very well fitted by such a function (R2 > 0.999). **Supporting Table S1:** Refractive index of the various water‐glycerol mixtures used throughout this study.

## Funding

This work was supported by the Interdisciplinary Institute HiFunMat (IdEx Unistra ANR‐10‐IDEX‐0002) and SFRI STRAT’US project (ANR‐20‐SFRI‐0012); CSC Graduate School of the University of Strasbourg, funded by the French National Research Agency (CSC‐IGS ANR‐17‐EURE‐0016); Ministry of Science and Education, Republic of Azerbaijan.

## Conflicts of Interest

The authors declare no conflicts of interest.

## Supporting information

Supplementary Material

## Data Availability

Data will be made available upon request to the corresponding authors.
