# Supplementary material for: Plasmonic Chiral Metasurfaces for Real‐Time Refractive Index Sensing and In Situ Monitoring of Thin Film Growth
Source: Small Sci. 2026 Mar 16;6(3):e202500568. doi: 10.1002/smsc.202500568 (PMC13097443; doi:10.1002/smsc.202500568)
Supplement: Supplementary file 1 — Supplementary Material [file SMSC-6-e202500568-s001.pdf]

Supporting Information

Plasmonic Chiral Metasurfaces for Real-Time Refractive Index Sensing and In Situ Monitoring of Thin Film Growth

Sevil Veysalova, Benjamin Boglio, François Courtier, Gero Decher, Olivier Felix* and Matthias Pauly**

1. Assembly of the flow cell

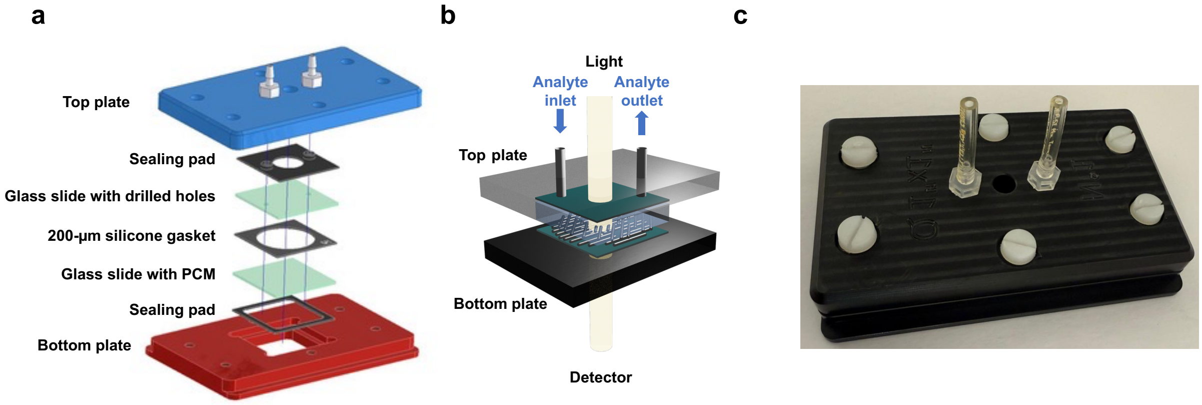

Figure S1. (a) *Exploded view of the different elements constituting the flow cell and its holder.* ***(b)*** *Scheme of the light path through the flow cell and plasmonic chiral metasurface.* ***(c)*** *Photo of the flow cell and holder.*

2. Refractive index of water-glycerol mixtures

The refractive index (RI) of water-glycerol mixtures was calculated from $n = A_n C + n_D^*$ where n is the RI of the mixture, $A_n \left(\frac{L}{mol}\right)$ is an experimental parameter which is $0.0105 \frac{L}{mol}$ for glycerol-water mixtures, C is the molar concentration of glycerol, and n_D^* is the refractive index of the solvent (1.333 for water).[1]

Table S1. Refractive index of the various water-glycerol mixtures used throughout this study

$m_{gly}(g)$	$m_{water}(g)$	$C_{gly}\left(\frac{mol}{L}\right)$	n
1.8387	98.5290	0.1997	1.3355
0.3325	9.9165	0.3546	1.3371
1.9256	48.4595	0.4182	1.3378
2.9900	47.6080	0.6494	1.3402
7.0462	94.3630	0.7651	1.3414
4.9800	46.0160	1.0816	1.3448
1.6554	10.6186	1.5052	1.3492
16.7381	86.6095	1.8176	1.3525
5.0147	20.9882	2.1782	1.3563
29.7411	76.2071	3.2296	1.3673
3.4432	8.2308	3.4036	1.3691
21.1639	33.0689	4.5964	1.3817
4.9000	5.1990	5.8349	1.3947
12.0018	10.3986	6.5163	1.4018
30.4501	25.6399	6.6131	1.4028
6.3446	3.5920	7.9486	1.4169
40.6056	17.5155	8.8187	1.4260

3. Refractive index sensing

a

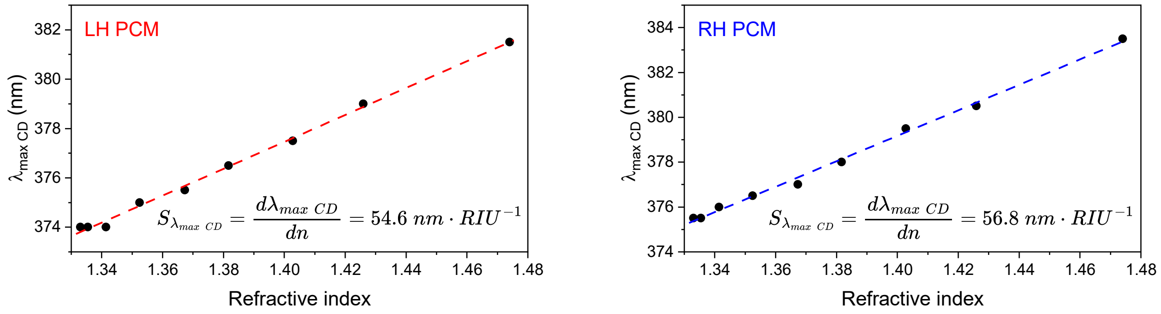

b

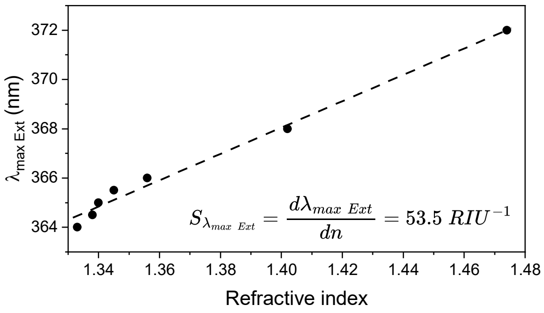

Figure S2. *Refractive Index Sensing based on the shift of the peak of **(a)** CD and **(b)** extinction spectra.*

To determine the most appropriate sensitivity based on CD changes at a constant wavelength, we calculated the sensitivity at different wavelengths and selected the one with the highest sensitivity (Figure S2). The highest sensitivity is observed at λ=365 nm, with an averaged value between the two enantiomorphs of 18700 $mdeg \cdot RIU^{-1}$.

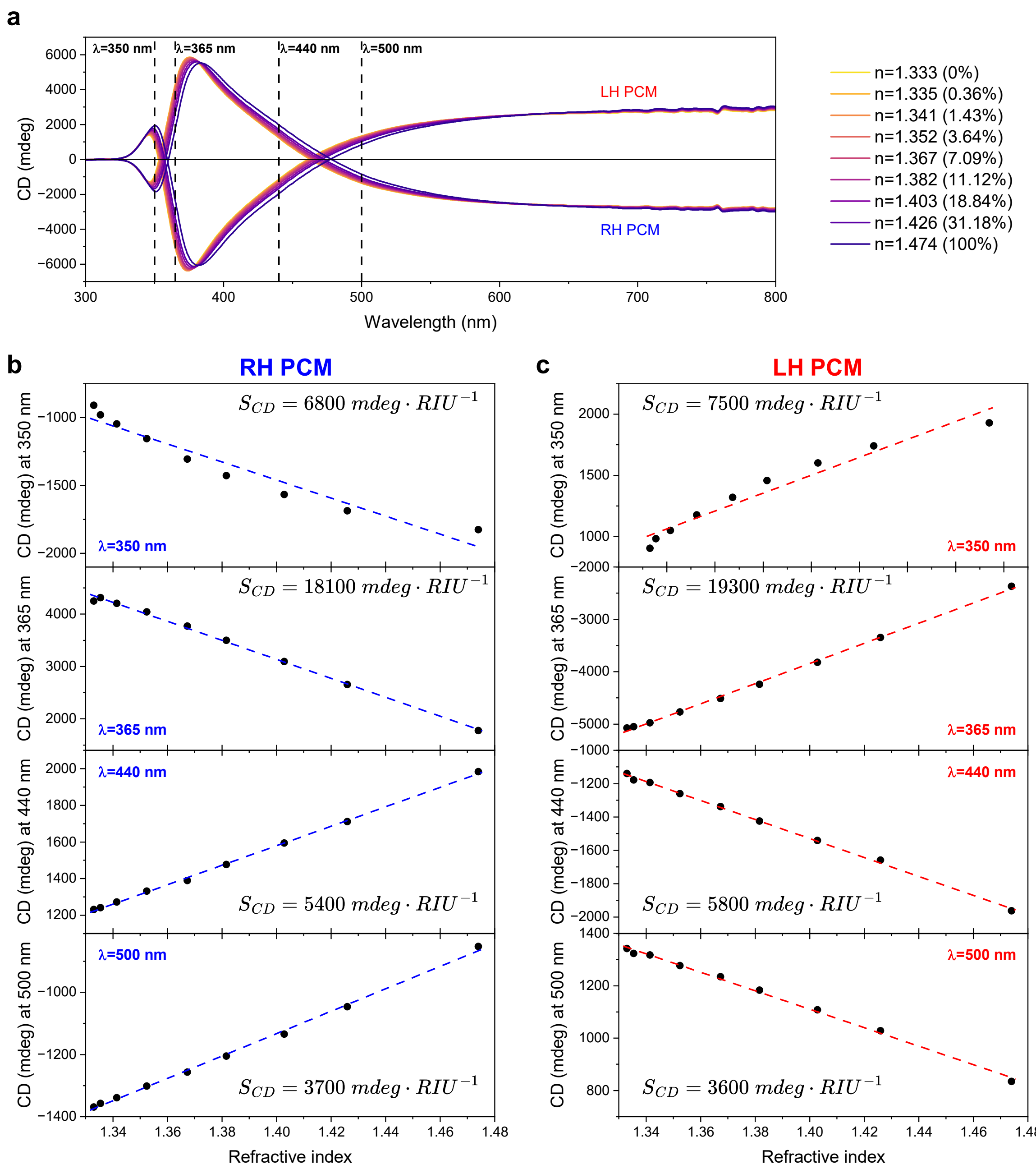

Figure S3. Refractive index sensing using CD. (a) *Circular dichroism (CD) spectra of right-handed and left-handed plasmonic chiral metasurfaces (PCMs) in contact with aqueous glycerol solutions of varying refractive index (RI).* ***(b, c)*** *CD intensity at λ=350 nm, λ=365 nm, λ=440 nm and λ=500 nm (from top to bottom) as a function of RI for (b) RH PCM and (c) LH PCM.*

We also conducted refractive index sensing experiment with a plasmonic chiral metasurface (PCM) constructed with a thicker electrolyte spacer between the two AgNW layers (Figure S3). In this experiment, we first deposited PEI/$(PSS/PAH)_5$/PSS/PEI on the substrate under the same experimental conditions as described previously, giving a total thickness of around 13 nm. After the deposition of a first AgNW monolayer by GIS, a thicker PEI/$(PSS/PAH)_{14}$/PSS/PEI interlayer was deposited and a second monolayer of AgNWs oriented at a −45° was added to

create a left-handed plasmonic chiral metasurface. For the refractive index sensing measurements, we again started with water and gradually increased the glycerol concentration. The sensitivity based on the CD value at a constant wavelength is measured as $S_{CD} \approx 2800\, mdeg \cdot RIU^{-1}$ at λ=355 nm and $S_{CD} \approx 2300\, mdeg \cdot RIU^{-1}$ at λ=388 nm, while the sensitivity based on the shift of the peak of the CD spectra is $S_{\lambda_{max}} \approx 66\, nm \cdot RIU^{-1}$.

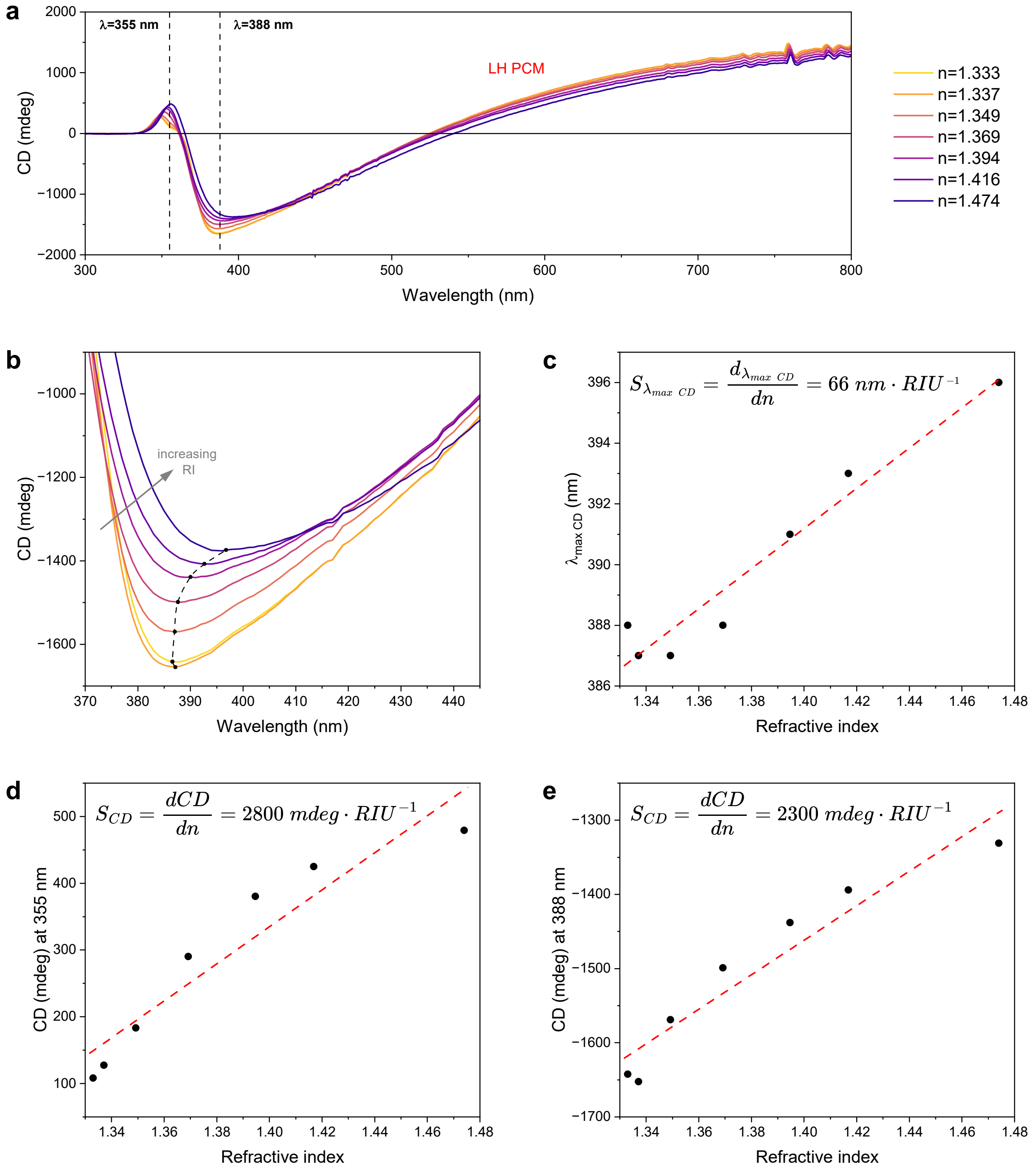

Figure S4. Refractive index sensing using CD spectra for a PCM with a thicker polyelectrolyte spacing between the AgNW layers. (a) *Circular dichroism (CD) spectra of left-handed plasmonic chiral metasurfaces in contact with aqueous glycerol solutions of varying refractive index (RI).* ***(b)*** *Magnified view of the CD spectra (a).* ***(c)*** $\boldsymbol{\lambda_{max\ CD}}$ *as a function of RI. CD intensity at* ***(d)*** *λ=355 nm and* ***(e)*** *λ=388 nm plotted as a function of RI for the left-handed PCM.*

4. 4×4 transfer-matrix simulations of the plasmonic chiral metasurfaces

The optical response of plasmonic chiral metasurfaces (PCMs) can be simulated using a 4×4 transfer-matrix formalism. The Python implementation *Pyllama* is employed here.[2] This formalism rigorously quantifies the change in amplitude and phase of light across each layer of a multilayer stack, assuming plane wave illumination, thus affording the reflection and transmission coefficients. Each layer is characterized, optically speaking, by two parameters: the thickness, and the wavelength-dependent complex refractive index. The method implicitly assumes that the layers are homogeneous and infinite in the x-y plane, with the z-axis being the axis of propagation.

The 4×4 matrix formulation is obtained by considering the electric and magnetic fields as vectors which encode the magnitude and phase of the fields along the Cartesian axes, i.e. $\vec{\boldsymbol{E}} = (E_x, E_y, E_z)$ and $\vec{\boldsymbol{H}} = (H_x, H_y, H_z)$. Combining this into one 6-dimensional vector, we arrive at a 6×6 matrix which describes the propagation of this vector across all layers. This 6×6 matrix stems from Maxwell's equations (specifically the curl equations), which after some algebra, and due to continuity constraints across layers, can be remodelled into a 4×4 matrix. In contrast, the typical 2×2 formulation, while less computationally expensive, does not allow for layers characterised by anisotropic refractive indices, which, in the 4×4 formalism, are expressed as tensors rather than scalars.

The AgNW layers behave as uniaxial birefringent slabs due to individual nanowire aspect ratio and GIS-induced alignment, making the 4×4 formalism necessary. The ordinary and extraordinary indices were previously measured by Hu *et al* and are employed here.[3]

The simulated stack attempts to replicates the experimental conditions (Figure S4):

- *Entry medium*: semi-infinite layer characterized by a refractive index varying from 1.333 to 1.474
- *Top AgNW (±45°)*: 30 nm thick, rotation of −45° and +45° for RH and LH samples, respectively
- *Middle spacer*: 13 nm polyelectrolyte layer with a refractive index that also varies from 1.333 to 1.474
- *Bottom AgNW (0°)*: 30 nm thick, oriented 0°
- *Bottom spacer*: 5 nm polyelectrolyte layer, constant refractive index of 1.465
- *Exit medium*: semi-infinite quartz layer, refractive index from Malitson.[4]

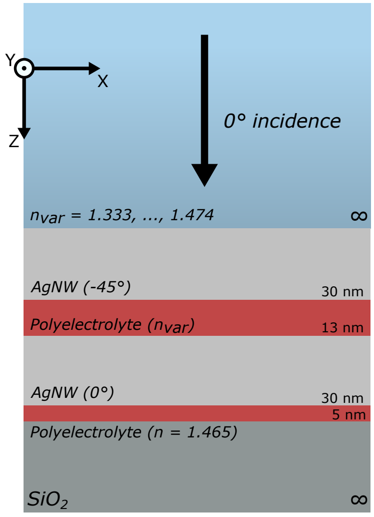

Figure S5. Schematic of the simulated multilayer stack*: aqueous/glycerol entry medium with varying refractive index (n_{var} = 1.333, ..., 1.474), oriented AgNW film (±45°, 30 nm), polyelectrolyte spacer (13 nm, n_{var}), oriented AgNW film (0°, 30 nm), polyelectrolyte spacer (5 nm, n = 1.465), and quartz substrate.*

All simulations are under normal incidence. Circular dichroism (CD) in millidegrees is calculated as:

$$CD_T = \log_{10}\frac{T_{RH}}{T_{LH}} \times 32980$$

Where $T_{LH/RH}$ are the transmittances for left- and right-handed light. This quantity does not consider the extinction due to reflection (and scattering more broadly), and therefore does not represent the true *losses* (absorption) of the system, i.e:

$$CD_A = \log_{10}\frac{(T_{RH} + R_{RH})}{(T_{LH} + R_{LH})} \times 32980$$

When working with molecules in solution, reflection is negligible, and the above formulations are equivalent. In the present case however, the metallic nature of the AgNW layers means strong reflection coefficients, and the CD based on transmittance, CD_T, is therefore the quantity that the commercial Jasco CD spectrometer measures, and is the simulated quantity presented below.

The CD of the modelled system is presented in Figure S5. Both qualitatively and quantitatively, the shape and magnitude of the CD spectra are reasonably well matched with the experimental case, with a peak CD value close to 5000 mdeg—though the simulated spectra are globally blue-shifted relative to the experimental results. Where the experimental and simulated values greatly differ however, is in the trend observed at the peak derivative value (365 nm in the experimental case, and 350 nm in the simulated case). Both trends are fitted with a linear equation, and while both slopes share the same sign, the magnitude of the slopes differ by a factor of ~20.

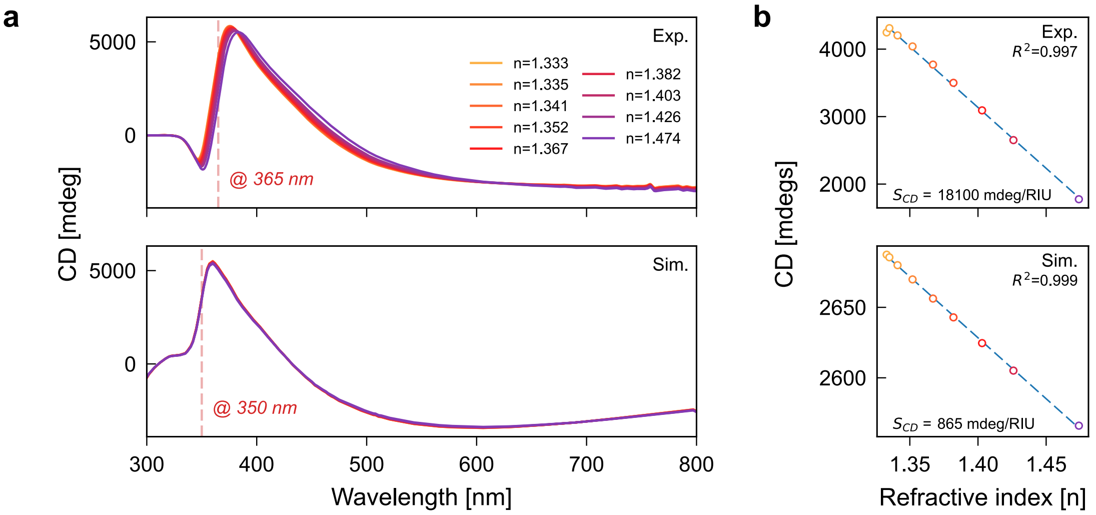

Figure S6. (a) *Experimental (top) and simulated (bottom) CD spectra for refractive indices from 1.333 (yellow) to 1.474 (purple). Dashed line indicates the wavelength used for sensitivity analysis (365 nm experimentally and 350 nm for the simulations) (b) CD at aforementioned wavelength versus refractive index, linear fits yield slopes with magnitudes of ~18100 mdeg·RIU^{-1} (exp., R^2 = 0.997) and ~865 mdeg·RIU^{-1} (sim., R^2 = 0.999).*

Although the simulated refractive-index sensitivity is more than two times lower than the sensitivity determined in the experiment, the 4×4 transfer-matrix model still captures the main CD features and their dependence on ambient refractive index. The quantitative discrepancies trace back to uncertainties in the AgNW films' effective refractive index—stemming from variations in nanowire dimensions and local packing density, both of which affect the effective permittivity and can vary with the surrounding refractive index—rather than any intrinsic limitation of the formalism. Therefore, despite these differences, the simulations remain an important tool for guiding the design of future sensors.

5. Real-Time Monitoring of Layer-by-Layer (LbL) Assembly via Microfluidics

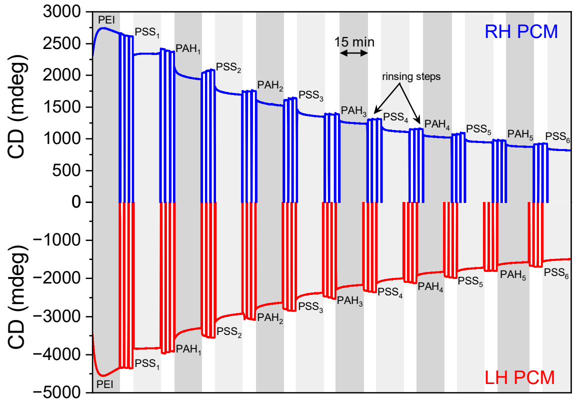

Figure S7. In situ assembly of polyelectrolyte multilayers in the flow cell with rinsing steps. *The CD is monitored at* $\lambda = 365$ nm *throughout the deposition of polyethyleneimine (PEI), poly(sodium 4-styrenesulfonate) (PSS), and poly(allylamine hydrochloride) (PAH).*

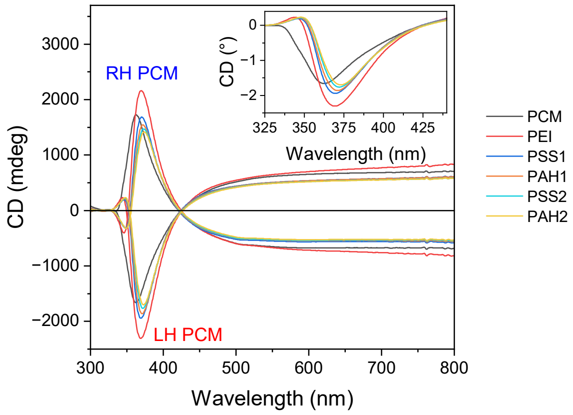

Figure S8. Circular Dichroism spectra of in situ assembly of polyelectrolyte multilayers in the flow cell. *The spectra labeled "PCM" is the one of the PCM in air before adding the polyelectrolyte layers, while the other spectra are measured after adsorption of the respective polyelectrolyte layers, the PCM still in contact with the polyelectrolyte solution. The inset shows a zoom of the CD spectra for LH PCM.*

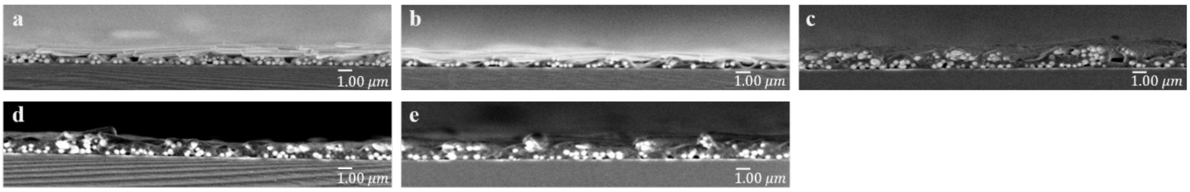

Figure S9. Cross-section SEM images *of* ***(a)*** *PCM,* ***(b)*** *PCM+PEI,* ***(c)*** *PCM+PEI/(PSS/PAH)*$_7$*,* ***(d)*** *PCM+PEI/(PSS/PAH)*$_{10}$*,* ***(e)*** *PCM+PEI/(PSS/PAH)*$_{15}$*.*

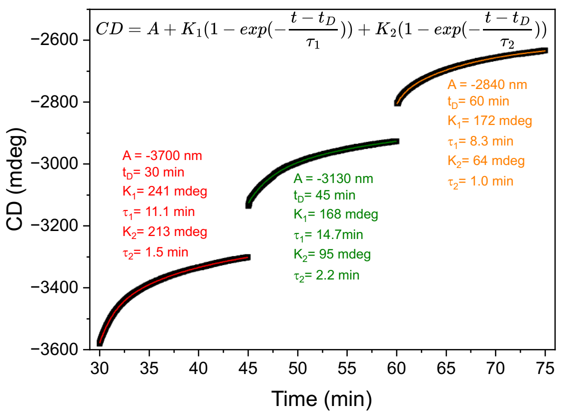

Figure S10. In situ assembly of polyelectrolyte multilayers in the flow cell without the rinsing steps. *The measured CD as function of time (black points) is fitted with a double exponential growth curve (colored line).*[1]

$$CD = A + K_1\left(1 - exp\left(-\frac{t - t_D}{\tau_1}\right)\right) + K_2\left(1 - exp\left(-\frac{t - t_D}{\tau_2}\right)\right)$$

for the first PAH layer (from 30 to 45 min), the second PSS layer (from 45 to 60 min) and the second PAH layer (from 60 to 75 min). The data are very well fitted by such a function (R^2>0.999).

To investigate the effect of adding polyelectrolyte layers on top of the plasmonic chiral metasurface (PCM), we prepared five samples on silicon wafers for cross-section imaging using scanning electron microscopy (SEM). We used spray-assisted layer-by-layer (LbL) deposition to construct the polyelectrolyte multilayer which is deposited between the two AgNW layers, but used dipping-assisted LbL deposition for the polyelectrolytes deposited above the PCM, mimicking the procedure used in the flow-cell. The samples are immersed for 15 minutes in the polyelectrolyte solution, followed by three rinsing steps of two minutes each with Milli-Q water. The first sample (Figure S9a) consisted of a PCM on which no polyelectrolyte layer is deposited. The second sample (Figure S9b) is a PCM with identical structure, which is covered with a PEI layer. The next samples are PCMs covered with polyelectrolyte multilayers of increasing thicknesses ($PEI/(PSS/PAH)_7$ in Figure S9c, $PEI/(PSS/PAH)_{10}$ in Figure S9d and $PEI/(PSS/PAH)_{15}$ in Figure S9e. Cross-section scanning electron microscope (SEM) images confirm that the structure of the PCMs remains unchanged upon the addition of the polyelectrolyte layers.

References

[1] F. Koohyar, A. A. Rostami, M. J. Chaichi, F. Kiani, *J. Solut. Chem.* **2011**, *40*, 1361.

[2] M. M. Bay, S. Vignolini, K. Vynck, *Comput. Phys. Commun.* **2022**, *273*, 108256.

[3] H. Hu, S. Sekar, W. Wu, Y. Battie, V. Lemaire, O. Arteaga, L. V. Poulikakos, D. J. Norris, H. Giessen, G. Decher, M. Pauly, *ACS Nano* **2021**, *15*, 13653.

[4] I. H. Malitson, *J. Opt. Soc. Am.* **1965**, *55*, 1205.
